# Fanconi anemia-associated mutation in RAD51 compromises the coordinated action of DNA-binding and ATPase activities

**DOI:** 10.1016/j.jbc.2023.105424

**Published:** 2023-11-02

**Authors:** Sijia Liu, Akira Shinohara, Asako Furukohri

**Affiliations:** Institute for Protein Research, Osaka University, Suita, Osaka, Japan

**Keywords:** DNA repair, Fanconi anemia, homologous recombination, RAD51, DNA binding protein

## Abstract

Fanconi anemia (FA) is a rare genetic disease caused by a defect in DNA repair pathway for DNA interstrand crosslinks. These crosslinks can potentially impede the progression of the DNA replication fork, consequently leading to DNA double-strand breaks. Heterozygous *RAD51-Q242R* mutation has been reported to cause FA-like symptoms. However, the molecular defect of RAD51 underlying the disease is largely unknown. In this study, we conducted a biochemical analysis of RAD51-Q242R protein, revealing notable deficiencies in its DNA-dependent ATPase activity and its ATP-dependent regulation of DNA-binding activity. Interestingly, although RAD51-Q242R exhibited the filament instability and lacked the ability to form displacement loop, it efficiently stimulated the formation of displacement loops mediated by wild-type RAD51. These findings facilitate understanding of the biochemical properties of the mutant protein and how RAD51 works in the FA patient cells.

The maintenance of genome integrity is crucial for ensuring survival and reproduction. To counteract the various genotoxic stresses from both endogenous and exogenous events, cells have evolved many types of DNA repair and damage tolerance mechanisms. The Fanconi anemia (FA) pathway is the one known to recognize and repair interstrand crosslinks (ICLs), a type of DNA lesion. FA was originally documented to be a rare hereditary disorder linked to genomic instability (1, 2). It is caused by a defect in a pathway found to be specialized for ICL repair. Most patients with FA have congenital anomalies such as short stature, thumb abnormalities, developmental irregularities, bone marrow failure, and a pronounced predisposition to cancer, notably acute myeloid leukemia (3, 4). The hallmark of cells derived from patients with FA is an increased cellular sensitivity to crosslinking agents such as mitomycin C (MMC) (5, 6). Presently, the FA pathway has been found to involve at least 23 genes, and mutations in any of which can give rise to FA disease due to their roles in DNA repair mechanisms responding to ICLs. An ICL lesion generated during the S phase can impede the replication fork progression but FANCM serves as a sensor for ICLs and forms an FA core complex with associated proteins, which in turn ubiquitinates the FANCI-FANCD2 heterodimer (ID2 complex) (7, 8). The ID2 complex activates endonucleases that unhook the ICL from one of the DNA strands. Translesion synthesis then bypasses the lesion, and the resultant double-strand break (DSB) undergoes repair through homologous recombination (HR) (2, 5, 9).

HR is essential for the accurate repair of DSBs to maintain genome stability. RAD51, a homolog of prokaryotic RecA, is the key recombinase carrying out homology search, strand invasion, and strand exchange between single-stranded DNA (ssDNA) and double-stranded DNA (dsDNA) during HR (10, 11, 12, 13). RAD51 forms a nucleoprotein filament on the 3′-overhang ssDNA generated at the DSB site for the strand exchange. The RAD51 filament searches for homologous dsDNA and interacts with it to form a joint molecule termed the displacement loop (D-loop) *via* DNA strand exchange. During this intricate process, RAD51 binds to both ssDNA and dsDNA through two disordered loops called L1 and L2 loops (14, 15, 16, 17). Possessing Walker A and B motifs, RAD51 displays an ATPase activity that is dramatically stimulated by the DNA binding (18, 19). Allosteric regulation of ATPase and DNA-binding activities through the interaction between RAD51-RAD51 protomers is predicted to control RAD51 filament dynamics in ssDNA (20).

Structural analysis of *E.coli* RecA has elucidated that binding of ssDNA stimulates its ATPase activity. The binding of RecA to ssDNA results in the movement of A148 toward the ATPase active site. Consequently, this movement repositions the catalytic residue E96 in proximity to the γ-phosphate of ATP at the active site and thereby stimulates ATP hydrolysis (17, 21). ATP binding at the interface between protomers stabilizes RAD51 filaments by increasing the cooperative assembly of RAD51 protomers on ssDNA rather than increasing the DNA-binding activity of monomeric RAD51 (22, 23, 24, 25). Insights from structural studies have further revealed that an ATP molecule is sandwiched between the ATPase motifs of two adjacent RAD51 protomers and serves as a glue to stabilize the interaction between these two protomers (15, 16, 17).

Although RAD51 has historically been recognized as a key recombinase in the strand exchange process during HR, four heterozygous RAD51 mutations—*RAD51-T131P*, *RAD51-A293T*, *RAD51-Q242R*, and *RAD51-A294T*—have recently been found to cause an FA-like phenotype, designated as FANCR (26, 27, 28, 29, 30). Patient-derived cells with these mutations showed hypersensitivity to crosslinking agents. Interestingly, cells derived from patients carrying *RAD51-T131P* mutation maintain the capacity for HR but exhibit severe deficiencies in ICL repair. This observation underscores that RAD51 has a distinct role within the FA pathway, in addition to its canonical role in HR (27). RAD51 is known to protect stalled replication forks from deleterious degradation mediated by MRE11 nuclease. MRE11 nuclease typically operates in DNA resection as MRE11-RAD50-NBS1 (MRN) complex during the initiation of HR (31, 32). FA pathway proteins, including FANCD2 and BRCA1/2, are also known to contribute to the protection of stalled forks from degradation by MRE11 (31). Investigations into RAD51-T131P and RAD51-A293T has suggested that the FA-associated mutant RAD51 may fail to protect stalled replication forks from uncontrolled degradation by MRE11 and DNA2 during ICL repair (26, 27). *In vitro* studies have proposed that RAD51-T131P and RAD51-A293T form unstable filaments with abnormal ATP binding, probably leading to a deficiency in fork protection (26, 27, 30).

While RAD51-T131P and RAD51-A293T have been analyzed in detail, RAD51-Q242R was solitary identified recently in a FA-like patient and has not yet been biochemically characterized (28). T131 is located inside the Walker A motif, and A293 is located adjacent to both the ATP-binding site and the L2 loop. Unlike these two mutated residues, Q242 is positioned away from the ATP-binding site but near the phosphate backbone of the incoming ssDNA and adjacent to the L1 loop for DNA binding (Fig. 1, *B* and *C*).

**Figure 1:**
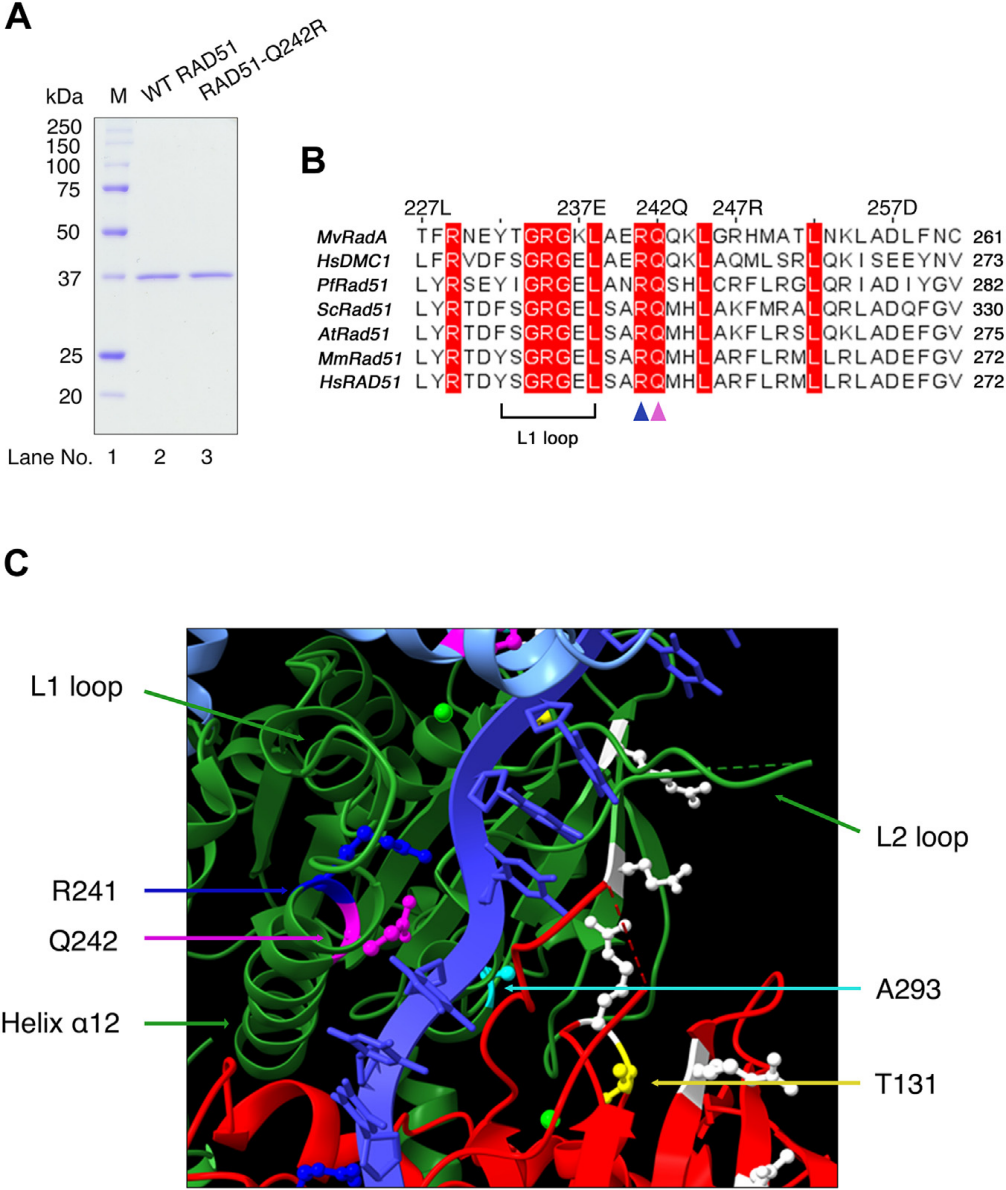
***RAD51-Q242R* mutation.***A*, gel image of human WT RAD51 and RAD51-Q242R. Purified proteins (0.5 μg) were loaded on a 12.5% SDS-PAGE and stained with Coomassie brilliant blue (CBB). M: molecular weight marker. *B*, sequence alignment of eukaryotic RAD51 and DMC1 representing the high conservation of two adjacent R241 and Q242 near the L1 loop (displayed using Jalview alignment editor (49)). *C*, structural model of human RAD51–ssDNA presynaptic complex (three protomers, shown as *pale blue*, *green*, and *red ribbons*, based on PDB 5H1B (15) and modified by using UCSF ChimeraX). Side chains of R241 and Q242 locating nearby DNA backbone are shown as *blue* and *magenta*, respectively. Side chain of T131 is shown as *yellow*, A293 is shown as *pale blue*, and residues included in site II binding (R130, R303 and K313 (42)) are shown as *white*. ssDNA, single-stranded DNA.

Here, we report that RAD51-Q242R protein, similar to the other two FA-RAD51 mutants, shows a severe defect in DNA-dependent ATPase and altered ATP-dependent DNA-binding activities. In contrast, unlike the other two mutants, RAD51-Q242R retains its DNA-binding activity and protects DNA against cleavage by MRN. Interestingly, although RAD51-Q242R shows the filament instability and cannot form a key HR intermediate, the D-loop, by itself, it can enhance the wild-type RAD51 activity to form the D-loop. Our results may provide new insights into the role of RAD51 in the FA pathway and how HR is maintained in FA-patient cells.

## Results

### RAD51-Q242R maintains ssDNA- and dsDNA-binding activity but does not show AMP-PNP-dependent stabilization of the RAD51 filament on DNA

**Figure 2:**
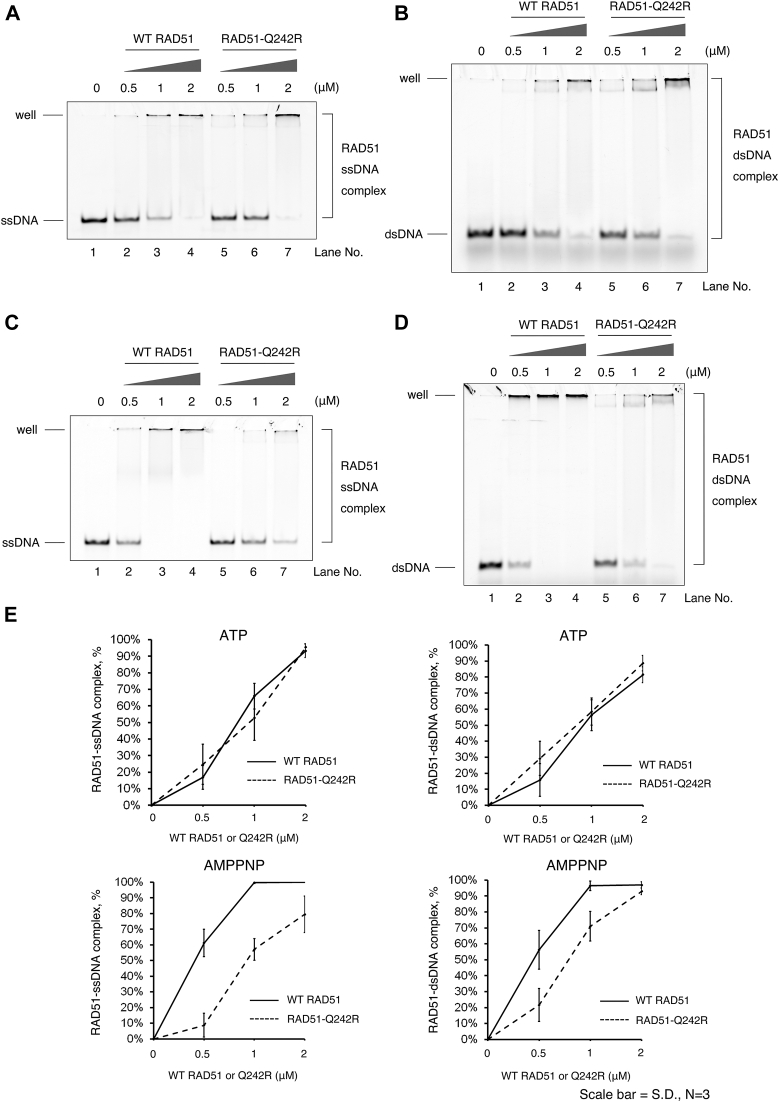
**Q242R retains ssDNA- and dsDNA-binding activities but has a defect in the ATP-dependent regulation of DNA-binding activity.***A–D*, DNA-binding assays using indicated concentrations of WT RAD51 and RAD51-Q242R for ssDNA (*A*) and dsDNA in the presence of ATP (*B*), ssDNA (*C*), and dsDNA (*D*) in the presence of AMP-PNP were carried out, respectively. The percentage of DNA–protein complexes relative to total DNA was calculated based on the quantification of the band intensities of unbound DNA, as shown in (*E*). dsDNA, double-stranded DNA; ssDNA, single-stranded DNA.

We purified human wild-type RAD51 and the FA-mutant RAD51-Q242R proteins (denoted as WT RAD51 and RAD51-Q242R, respectively) to near homogeneity (Fig. 1*A*). RAD51 has two conserved DNA-binding motifs, L1 and L2 loops (14). Two adjacent residues, R241 and Q242, downstream of the L1 loop are highly conserved among eukaryotic RAD51 and its meiosis-specific homolog, DMC1 (33) (Fig. 1*B*). While R241 is likely involved in interaction with the phosphate backbone of DNA (15) (Fig. 1, *B* and *C*), the role of Q242 remain unexplored. To investigate whether the Q242R mutation has impact on the ssDNA- and dsDNA-binding activities of RAD51, we carried out gel shift assays using short, linear ssDNA or dsDNA (Fig. 2).

Two previously examined mutant proteins associated with FA, RAD51-T131P, and RAD51-A293T displayed significant reduction in their abilities to bind to both ssDNA and dsDNA in gel shift assays (26, 27, 30). In contrast, a comparable level of DNA binding was observed for both WT RAD51 and RAD51-Q242R using ssDNA and dsDNA as a substrate in the presence of ATP (Fig. 2, *A* and *B*), suggesting that RAD51-Q242R retains nearly normal levels of ssDNA- and dsDNA-binding activities. The salt concentrations within the range of 0 to 150 mM KCl had minimal impact on the ssDNA-binding activity of both WT RAD51 and RAD51-Q242R (Fig. S4).

Multiple RAD51 molecules bind to DNA to form a nucleoprotein filament, which is known to be the active form of RAD51 recombinase. ATP binding at the protomer–protomer interface promotes filament stabilization and ATP hydrolysis destabilizes the RAD51-ssDNA filament (17, 22, 23, 24, 25). As reported previously, in the presence of a nonhydrolyzable ATP analog, AMP-PNP, the amount of ssDNA–RAD51 complex increased for RAD51 WT (at 1 μM RAD51, ∼66% of ssDNA formed the complex with ATP and ∼100% with AMP-PNP, compare Fig. 2, *A* and *C*, see Figs. 2*E* and S1*C*). In contrast, the increase of the amount of the ssDNA-RAD51-Q242R complex upon the addition of AMP-PNP was comparatively less than that observed with WT RAD51 (at 1 μM RAD51-Q242R, ∼50% of ssDNA formed the complex with ATP and ∼55% with AMP-PNP), suggesting that the inhibiting ATP hydrolysis does not greatly enhance the stability of the RAD51-Q242R-ssDNA filament. This trend was similarly observed for dsDNA (Figs. 2, *B* and *D* and S1*D*). The results imply that RAD51-Q242R may have a defect in ATPase activity or ATP-regulating dynamics of the protein on DNA.

### RAD51-Q242R retains a basal level of ATPase activity but lacks DNA-dependent ATPase activity

**Figure 3:**
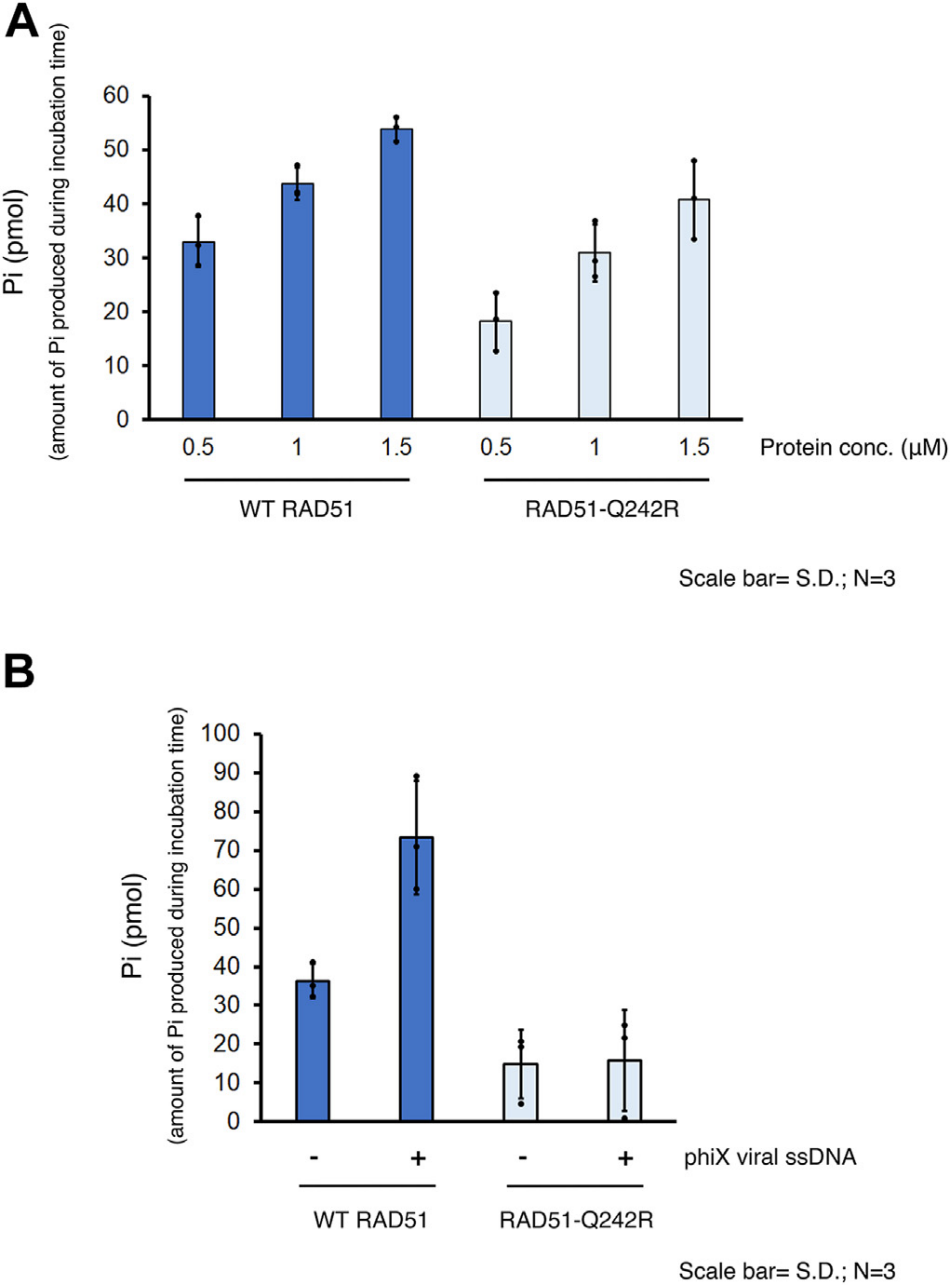
**RAD51-Q242R retains a basal level of ATPase activity but lacks DNA-dependent ATPase activity.***A*, ATPase activity was tested using the indicated concentrations of WT RAD51 and RAD51-Q242R by measuring the amount of free phosphate produced by ATP hydrolysis during a 20-min incubation in the absence of DNA. *B*, the amount of free phosphate was measured using 0.5 μM WT RAD51 or RAD51-Q242R in the presence or absence of phiX ssDNA after a 40-min incubation. ssDNA, single-stranded DNA.

Two other known FA-related mutations in RAD51, T131P, and A293T are predicted to be located near the interface of RAD51 protomers forming the ATPase active center (Fig. 1*C*) (34), and RAD51-T131P and RAD51-A293T proteins showed abnormal ATPase activities (26, 27, 30). Given that the Q242R substitution is located on the protein surface, positioned opposite to the ATP-binding site, we speculated that Q242R might not affect the ATPase activity. As expected, RAD51-Q242R showed a basal ATPase activity in the absence of DNA (Fig. 3*A*), although it is slightly lower than that of WT RAD51, showing that the mutant protein retains its capacity for both ATP binding and hydrolysis. The ATPase activity of WT RAD51 was greatly increased by the addition of phiX174 viral ssDNA as previously reported (18, 19, 35). Interestingly, the RAD51-Q242R mutant protein did not display any increase in ATPase activity when ssDNA was added, showing that the mutant protein lacks the DNA-dependent ATPase activity (Fig. 3*B*).

### RAD51-Q242R stably binds to ssDNA with altered filament stability

**Figure 4:**
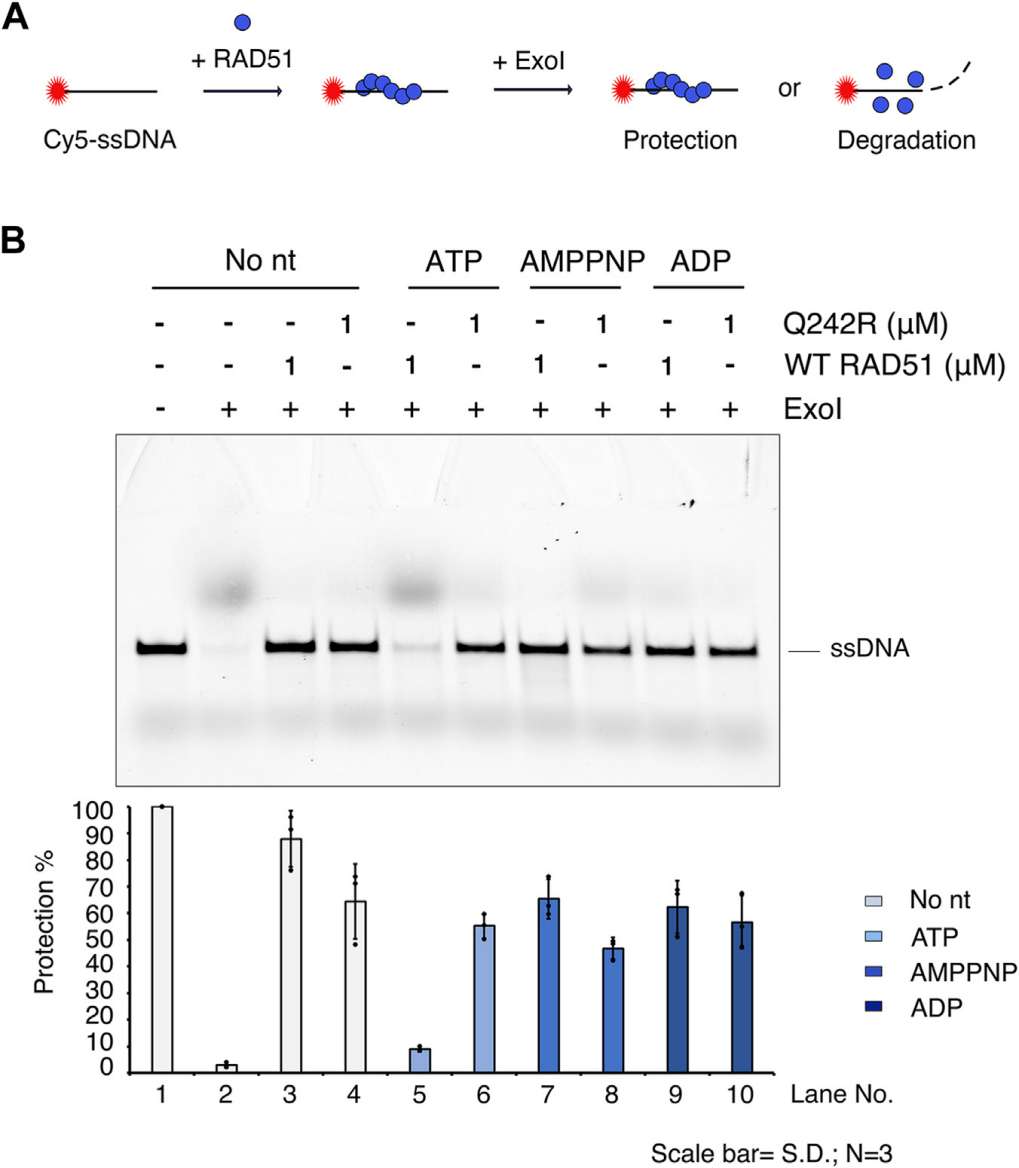
**RAD51-Q242R retains an ability to protect ssDNA from nuclease attack but shows a defect in ATP hydrolysis–dependent dissociation from ssDNA.***A*, reaction scheme of nuclease protection assay. ExoI was added to preformed RAD51-ssDNA nucleoprotein filament. Stable RAD51-ssDNA filament protects ssDNA, while DNA is cleaved by ExoI if RAD51 temporarily dissociates from ssDNA during the incubation. *B*, the effect of nucleotides on the stability of WT RAD51 and RAD51-Q242R. The reactions were carried out at indicated concentrations of proteins in the presence or absence of 2 mM ATP, AMP-PNP, and ADP. The percentage of intact DNA against total DNA was calculated based on the quantification of band intensities of intact DNA bands and is shown below the gel image. MRN, MRE11-RAD50-NBS1; ssDNA, single-stranded DNA.

Our findings indicate that RAD51-Q242R can form a complex with ssDNA but lacks DNA-dependent ATPase activity, which is known to destabilize the RAD51-ssDNA filaments (Figs. 2 and 3). This leads us to speculate that RAD51-Q242R might have a deficiency in ATP hydrolysis–induced dissociation from ssDNA, subsequently affecting the stability of the RAD51 filament. To investigate this, we conducted a nuclease protection assay to assess whether this substitution altered the stability of the RAD51–ssDNA filament in the presence of ATP (Fig. 4).

In this assay, a saturated amount of RAD51 was incubated with 5′–Cy5-labeled 90-mer ssDNA to form the nucleoprotein filaments in the presence or absence of ATP or AMP-PNP. The protein–DNA complex was further incubated with a bacterial 3′-to-5' exonuclease, ExoI, for 10 min. If the RAD51–ssDNA complex remained stable, RAD51 would protect the ssDNA substrate from ExoI digestion, while the DNA would undergo digestion if RAD51 temporarily dissociated from ssDNA during incubation (Fig. 4*A*). We observed that the protection by WT RAD51 was less pronounced in the presence of ATP compared to when ATP was absent or replaced with ADP (Fig. 4*B*, compare lanes 5 and 3, 9). This suggests that ATP hydrolysis actively destabilizes RAD51 nucleoprotein filaments as reported previously (25). Consistent with this, WT RAD51 became capable of protecting ssDNA from ExoI in the presence of AMP-PNP. In contrast, RAD51-Q242R provided ssDNA protection even in the presence of ATP, as without ATP or with AMP-PNP. This finding signifies that ATP hydrolysis–dependent disassembly and consequently RAD51 filament dynamics are greatly hindered by the Q242R substitution in RAD51, possibly due to its impaired DNA-dependent ATPase activity.

### RAD51-Q242R showed DNA protection ability against MRN complex

**Figure 5:**
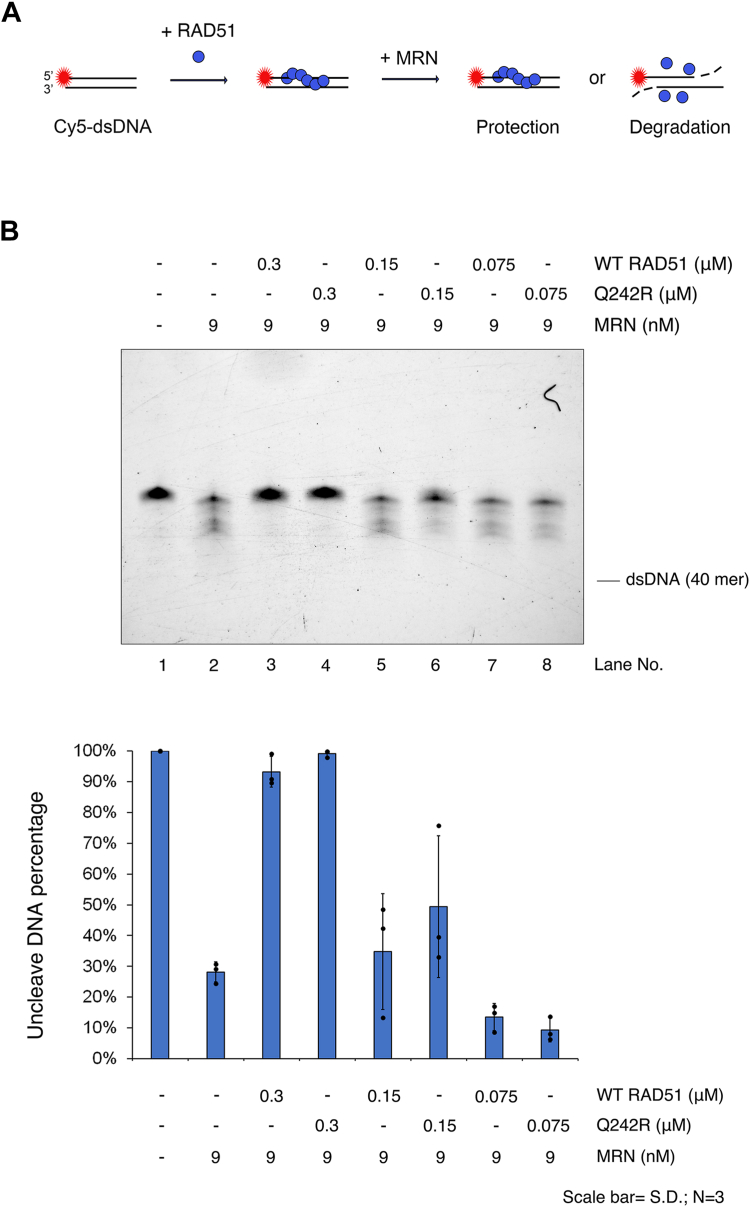
**RAD51-Q242R protects dsDNA against the cleavage of MRN similar to WT RAD51.***A*, reaction scheme of nuclease protection assay using MRN. *B*, decreasing concentration (0.3–0.075 μM) of WT RAD51 or Q242R were preincubated with 40-mer dsDNA for 5 min, and MRN was then added into indicated lanes at a final concentration of 9 nM and further incubated for 30 min. The percentage of intact DNA against total DNA was calculated based on the quantification of band intensities of intact DNA bands and is shown (bottom). dsDNA, double-stranded DNA; MRN, MRE11-RAD50-NBS1.

Previous studies have shown that RAD51-T131P and RAD51-A293T form unstable filaments and thus show their impaired ability to protect DNA from MRE11 nuclease (26, 36). MRE11 is the 3′–5' exonuclease subunit of the MRN complex, which is responsible for the degradation of nascent DNA strands at the stalled replication fork (31, 32). Given that RAD51-Q242R maintains its DNA-binding capability and can protect ssDNA from bacterial ExoI, as well as wild-type does, we hypothesized that RAD51-Q242R might also possess the ability to protect DNA from the MRN complex. We observed that WT RAD51 successfully protect dsDNA from the MRN complex, even in the presence of ATP. As anticipated, RAD51-Q242R exhibited protection from the MRN complex at a level comparable to that of WT RAD51 (Fig. 5).

### RAD51-Q242R lacks the strand exchange activity and releases ssDNA in the presence of competitor DNA

Although the association and dissociation of RAD51 with ssDNA are regulated by ATP binding and hydrolysis, the precise function of this protein dynamics during HR has not been fully understood. Binding of ATP to RAD51 is required for DNA pairing between homologous DNA strands; however, ATP hydrolysis is not required for the pairing reaction (25, 37, 38, 39). Rather, the prevention of ATP hydrolysis stabilizes the presynaptic filaments and stimulates the formation of a recombination intermediate called the D-loop. ATP hydrolysis by RAD51 destabilizes the filament and stimulates the turnover of RAD51, which is proposed to participate in the later step of the strand exchange process for efficient HR in cells (21, 25, 40).

**Figure 6:**
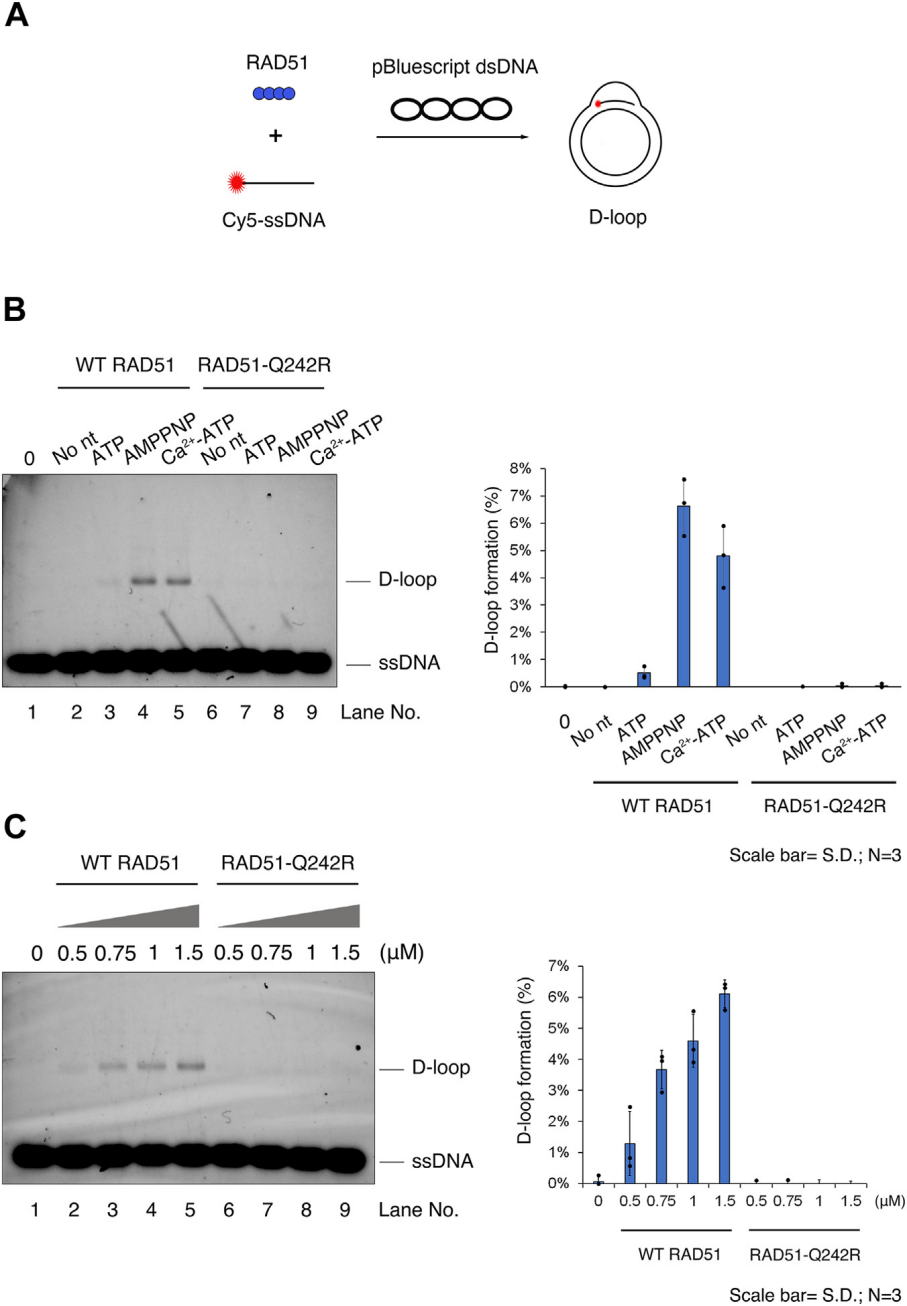
**RAD51-Q242R lacks D-loop formation ability.***A*, reaction scheme of the D-loop assay with Cy5-labeled ssDNA and supercoiled pBluescript plasmid DNA. *B*, D-loop assay was carried out using 1.5 μM WT RAD51 or RAD51-Q242R in the presence or absence of ATP or AMP-PNP, together with Mg^2+^ or Ca^2+^ ions. *C*, increasing concentrations of WT RAD51 or RAD51-Q242R were used in the D-loop assay in the presence of AMP-PNP and Mg^2+^ ions. The percentage of D-loop formation was quantified from band intensities of D-loop products of each sample compared to the band intensity of free ssDNA (corresponds to lane 1) are shown (*right*). D-loop, displacement loop; dsDNA, double-stranded DNA; ssDNA, single-stranded DNA.

In our nuclease protection assay, RAD51-Q242R demonstrated better protection of ssDNA compared to WT RAD51 in the presence of ATP (Fig. 4, compare lanes 5 and 6), implying that the RAD51–Q242R–ssDNA complex may exhibit increased stability compared to that of WT RAD51 under this condition. Consequently, we postulated that RAD51-Q242R might facilitate D-loop formation more efficiently than the wild-type protein in the presence of ATP. As reported, small amounts of D-loop products were formed by the addition of WT RAD51 in the presence of ATP, but D-loop formation was significantly stimulated when ATP hydrolysis was inhibited through the addition of Ca^2+^ or employing AMP-PNP (Fig. 6*B*). Contrary to our expectations, our results demonstrated that D-loop formation did not occur with RAD51-Q242R either in the presence of ATP, Ca^2+^, or AMP-PNP. The amounts of D-loop products increased with increasing concentrations of WT RAD51 in the presence of AMP-PNP, whereas RAD51-Q242R failed to mediate D-loop formation even at the highest concentration (3 μM) (Figs. 6*C* and S2*A*). Even when ATP/Mg^2+^ was combined with Ca^2+^ to stimulate D-loop formation, RAD51-Q242R could not effectively mediate this process, indicating that RAD51-Q242R lacks DNA-pairing activity (Fig. S2*B*).

In the strand pairing/exchange reaction, the RAD51-ssDNA nucleoprotein filament interacts with its homologous dsDNA to form a ternary complex of RAD51-ssDNA with dsDNA, then the exchange between homologous DNA strands occurs to form the D-loop (41). For the initial binding to ssDNA, RAD51 associates with ssDNA at the primary DNA-binding site composed of L1 and L2 loops (site I). The search and capture of dsDNA by RAD51–ssDNA complexes is mediated by a secondary DNA-binding site (site II), which is composed of positively charged amino acids located away from the site I (Fig. 1*C*) (42, 43). The affinity of site II for DNA is believed to be much weaker than that of site I (43); however, the substitution of amino acids at site II abolishes the strand-exchange ability of RAD51 without affecting its ability to bind to the ssDNA, indicating the essential role of site II in DNA binding in HR process (41, 43).

**Figure 7:**
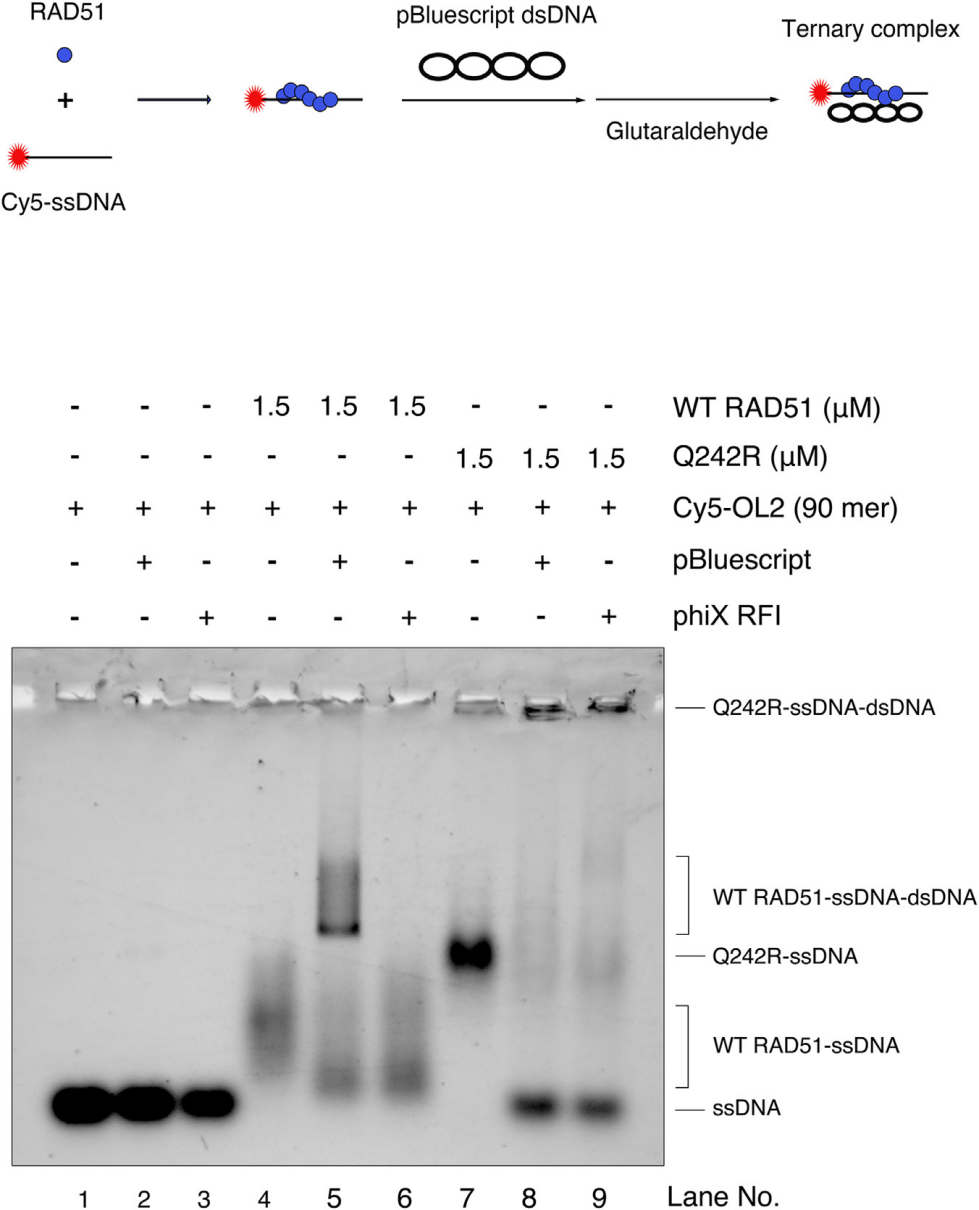
**RAD51-Q242R releases ssDNA in the presence of competitor dsDNA.** Ternary complex formation assay. The reaction mixtures were prepared using 1.5 μM WT RAD51 or RAD51-Q242R and 175 μM (in nt) pBluescript or phiX RFI as in the D-loop assay. The ternary complex was fixed by the addition of glutaraldehyde as shown in the scheme of the assay. The slower migrating band corresponding to the ternary complex was observed only when homologous dsDNA was used (compare lanes 5 and 6). Faster migrating, free ssDNA appeared when homologous or nonhomologous dsDNA was added into the reaction with Q242R (lanes 8 and 9). D-loop, displacement loop; dsDNA, double-stranded DNA; ssDNA, single-stranded DNA.

Although Q242 is situated in close proximity to site I, our gel shift assays have revealed that RAD51-Q242R is capable of forming complexes with both ssDNA and dsDNA, similar to WT RAD51, suggesting that the DNA-binding activity at the site I may remain largely intact in RAD51-Q242R (Fig. 2). Thus, we hypothesized that the RAD51-Q242R filament might not effectively capture donor dsDNA, primarily due to an impaired site II DNA binding resulting from the mutation. To test this hypothesis, we tested whether the ternary complex (RAD51–ssDNA–dsDNA complex) could be formed *via* the glutaraldehyde cross-linking experiment, using the same conditions employed for the D-loop formation assay. We used AMP-PNP, which stabilizes the RAD51 filaments for the D-loop formation. A smeared band corresponding to the RAD51–ssDNA complex was observed in the control reaction with WT RAD51, when no homologous dsDNA was added (Fig. 7, lane 4). A discrete band of the RAD51–Q242R–ssDNA complex migrating slower than that of WT RAD51-ssDNA was observed under the same conditions, suggesting that RAD51-Q242R efficiently bound to ssDNA compared to WT RAD51 under these conditions (Fig. 7, lane 7). When the dsDNA with a homologous sequence was added to the reaction with WT RAD51, a slow-migrating band of the ternary complex, likely WT RAD51-ssDNA-dsDNA, was observed. When nonhomologous dsDNA was added, the ternary complex band was not observed, but smeared bands corresponding to RAD51-ssDNA were observed (Fig. 7, lanes 5 and 6). Proteinase treatment of the reaction revealed D-loop products only when homologous dsDNA was added, suggesting that the WT RAD51–ssDNA–dsDNA complexes had a stable D-loop (Fig. S3*A*). However, the band corresponding to the ternary complex did not appear when RAD51-Q242R was used (Fig. 7, lanes 8 and 9), and the RAD51–Q242R–ssDNA complex almost disappeared when either homologous or nonhomologous dsDNA was added. Unexpectedly, a significant amount of free ssDNA was produced instead of the ternary complex, suggesting that RAD51-Q242R was bound to dsDNA while releasing ssDNA. This massive release was also evident after the addition of heterologous phiX ssDNA, suggesting that the RAD51-Q242R presynaptic filament at site I was highly unstable in the presence of a high concentrations of competitor DNAs (Fig. S3*B*).

### RAD51-Q242R does not dominantly inhibit the strand-exchange reaction by WT RAD51 and assists WT RAD51 to form a D-loop at a high concentration

**Figure 8:**
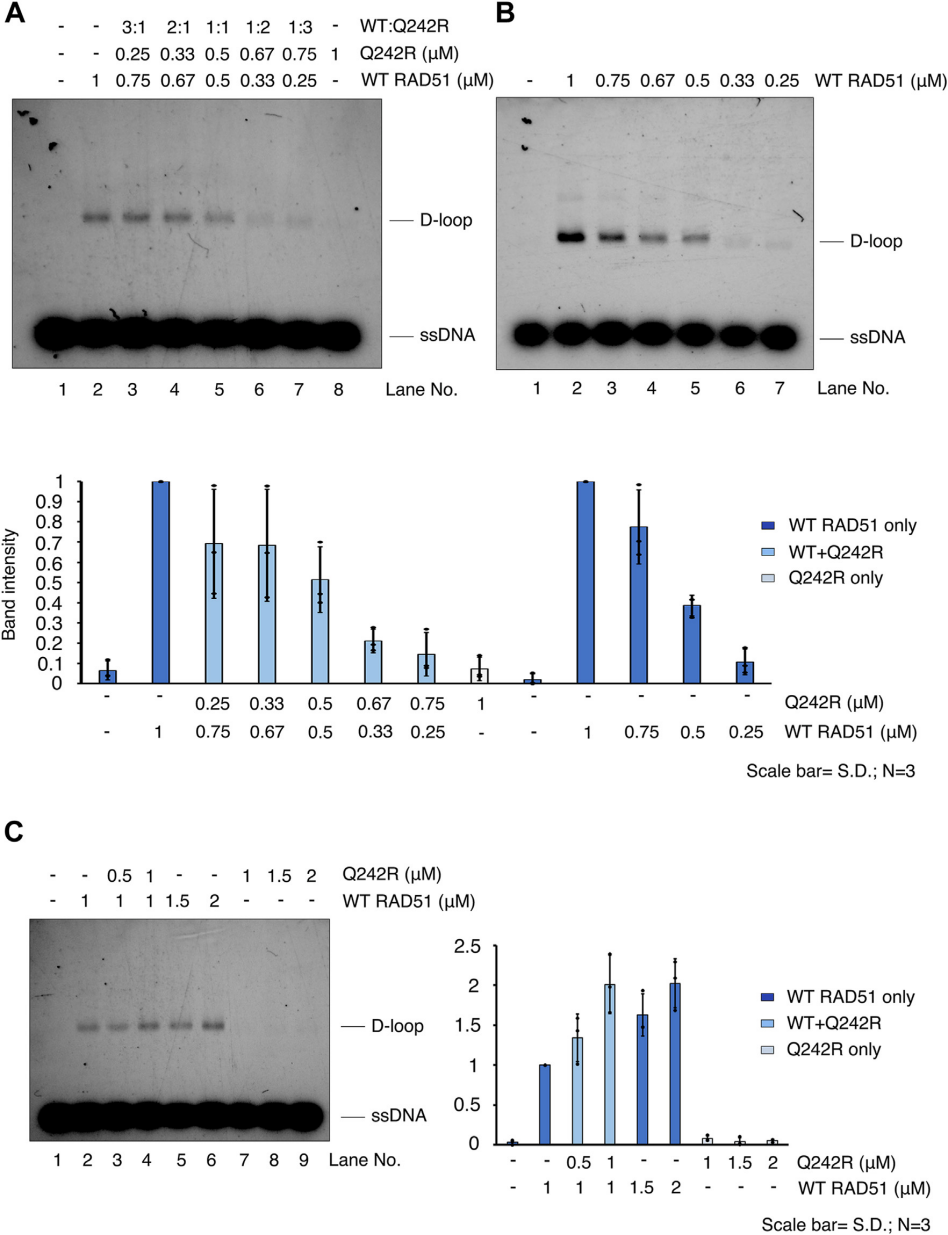
**RAD51 Q242R helps the strand-exchange reaction by WT RAD51 at a high concentration.***A*, indicated concentrations of WT RAD51 and RAD51-Q242R were mixed in D-loop assay. The ratios of two proteins are shown above the image. *B*, control reactions carried out only with WT RAD51 under the same conditions. The relative band intensities of D-loop products of each sample compared to that at 1 μM WT RAD51 (lane 2 in each gel image) are shown (bottom). *C*, various concentrations of RAD51-Q242R (0, 0.5, 1 μM, lanes 2–4) were added to the constant concentration of WT RAD51 (1 μM). Total RAD51 concentrations of lanes 3 and 4 are the same as those of the control reaction with WT RAD51 only in lanes 5 and 6 (1.5 and 2 μM). The relative band intensities of D-loop products of each sample compared to that at 1 μM WT RAD51 (corresponds to lane 2) are shown (*right*). D-loop, displacement loop; ssDNA, single-stranded DNA.

The heterozygous *RAD51-Q242R* mutation in RAD51 causes a high frequency of chromosomal breaks induced by MMC in patient cells, suggesting that RAD51-Q242R interferes with the function of WT RAD51 in a predominantly negative manner *in vivo* (28). To test if RAD51-Q242R dominantly inhibits the strand-exchange reaction by WT RAD51, we used both WT RAD51 and RAD51-Q242R at different ratios with the total RAD51 protein concentration of 1 μM in the D-loop assay. Increasing the percentage of RAD51-Q242R decreased the number of D-loop products (Fig. 8*A*). However, comparing them to the amounts in control reactions with only WT RAD51 at the same concentrations (1–0.25 μM, Fig. 8*B*), the amounts of D-loop products were not significantly reduced, suggesting that RAD51-Q242R does not inhibit WT RAD51 by out competing binding of WT RAD51 to ssDNA or by forming a mixed filament of both WT and RAD51-Q242R which is incapable of the strand-exchange reaction. Unexpectedly, the opposed effect of Q242R was observed at higher concentrations between 1 and 2 μM of total RAD51 proteins (Fig. 8*C*). The addition of RAD51-Q242R helped the formation of D-loop products by WT RAD51; the addition of 1 μM of RAD51-Q242R to 1 μM WT RAD51 produced a similar amount of D-loop product at 2 μM of WT RAD51 (Fig. 8*C*, compare lanes 4 and 6). We speculate that the mixed filament is formed by the rising concentration of RAD51 and that at this ratio, the mixed RAD51 filament is as capable of the strand-exchange reaction as the WT RAD51 filament is. In the reported RAD51-related FA patient cells, it is speculated that the FA pathway is hindered, but HR activity is maintained to some extent as RAD51-T131P expressing cells show HR proficiency (27). Our data suggest that the patient cells carrying the *RAD51-Q242R* mutation may also retain HR proficiency by forming a mixed filament of wild-type and mutant RAD51.

## Discussion

To investigate the causal mechanisms of RAD51-related FA disease, we biochemically characterized the RAD51-Q242R protein which has only been previously reported in a patient with FA. We observed that this mutant protein retained basal levels of DNA-binding and ATPase activities. However, its DNA-dependent ATPase activity was significantly impaired, and the protein dynamics on ssDNA were greatly altered, as the mutant protein did not exhibit ATP hydrolysis–dependent dissociation ability, suggesting that the mutation impairs the coupling of ATP binding/hydrolysis and DNA binding/dissociation. This is interesting because two other reported FA mutants, RAD51-T131P and RAD51-A293T, also showed similar defects in ATP- and DNA-dependent regulation of protein activities (26, 27, 30). It is possible that these common defects contribute to the FA-related phenotypes in patient cells, but currently the role of the coordination between ATPase- and DNA-binding activities in the FA pathway is largely unclear. The DNA-binding reaction of RAD51 is not a simple process of two biomolecules; rather, it involves multiple reactions of DNA binding/dissociation controlled by ATP binding/hydrolysis. This coordination may be important for maintaining proper stability of RAD51 filaments to protect the genome, which the mutation may disturb.

On the other hand, unlike RAD51-T131P and RAD51-A293T, which showed significantly weaker DNA-binding activities than did WT RAD51, RAD51-Q242R showed a level of DNA-binding activity comparable to that of the wild-type protein in the gel-shift assay. Nuclease protection assays also showed that RAD51-Q242R was capable of preventing DNA cleavage by bacterial ExoI and MRN, demonstrating that RAD51-Q242R can bind both ssDNA and dsDNA, similar to WT RAD51 (Figs. 4 and 5). In the presence of ATP, only WT RAD51 showed weaker protection of ssDNA against ExoI but could protect dsDNA against MRN under similar conditions, probably because WT RAD51 dissociates from ssDNA in an ATP-hydrolysis dependent manner. In the RAD51–ssDNA complex, two adjacent amino acids R241 and Q242, are close to the DNA backbone, and their side chains are located near the phosphate groups of DNA (Fig. 1*C*). In the RAD51-Q242R mutant protein, changing glutamine to arginine may rather facilitate DNA binding, as the positively charged guanidinium group of arginine can interact with the phosphate group by electrostatic attraction or hydrogen bond formation. In fact, the formation of ssDNA–protein complexes seems to be more efficient for RAD51-Q242R than WT RAD51 when the dissociation was inhibited by crosslinking with glutaraldehyde (Fig. 7, compare lanes 4 and 7), implying that RAD51-Q242R may bind to ssDNA faster than WT RAD51. Because the final amount of the DNA–protein complex seemed to be similar between WT RAD51 and RAD51-Q242R in the DNA-binding assay (Fig. 2*E*), RAD51-Q242R probably also dissociated from ssDNA faster than WT RAD51. Consistent with our speculation, we observed that RAD51-Q242R released ssDNA and was captured by a competitor DNA much faster than WT RAD51 in the ternary complex formation assay (Fig. 7). RAD51-Q242R may thermodynamically bind to DNA without proper filament nucleation. We speculate that this instability of the presynaptic complex is the main cause of the impaired D-loop formation ability of RAD51-Q242R. Future structural analysis is needed to determine whether Q242R cooperatively binds to ssDNA and forms a stable nucleoprotein filament structure or if it only randomly binds to ssDNA.

We observed that RAD51-Q242R lacks the ability to form D-loop products, similar to RAD51-T131P (27). A dominant negative-like behavior inhibiting WT RAD51 forming a D-loop has been reported for RAD51-T131P *in vitro*. Although RAD51-Q242R is also thought to dominantly inhibit the function of WT RAD51 *in vivo* because MMC sensitivity has been reported in patient cells harboring a heterozygous *RAD51-Q242R* mutation (28), the presence of RAD51-Q242R did not negatively interfere with WT RAD51 in our biochemical assays. We speculate that RAD51-Q242R may have the ability to form a functional filament with WT RAD51, which retains the ability to form a D-loop, as RAD51-Q242R has a basal DNA-binding activity. Cells with heterozygous *RAD51-T131P* mutations are HR proficient while severely affecting ICL repair because of the low ratio of mutant to WT RAD51 in patient cells (27). Our findings raise the possibility that similar to T131P patient cells, HR may also be proficient, even if both WT RAD51 and RAD51-Q242R are expressed in Q242R patient cells.

Currently, it is unclear how the function of WT RAD51 is predominantly inhibited by RAD51-Q242R to induce the FA phenotype. RAD51 is reported to play an HR-independent role in protecting nascent DNA at the replication fork when DNA replication is perturbed (42). In cells with T131P RAD51 mutations, DNA2-mediated degradation results in RPA phosphorylation and the accumulation of ssDNA following MMC treatment, suggesting that the mutant form of the protein dominantly affects the protective role of the stalled fork (27). RAD51 has also been reported to protect nascent DNA from nucleolytic degradation by MRE11 when replication fork progression is inhibited (31, 32). A recent *in vitro* study showed that RAD51’s dsDNA binding mechanically prevents MRE11-, DNA2-, and EXO1-dependent degradation, which is stimulated by the presence of the C-terminal RAD51 segment of BRCA2 (36, 44). Both RAD51-T131P and RAD51-A293T showed weak DNA protection against MRE11 or MRE11-RAD50 compared to WT RAD51 (26, 36). Like the other two mutants, one possibility is that the stability of WT RAD51 filament formed in the presence of RAD51-Q242R may not be stable enough to protect the replication fork in FA pathway. However, RAD51-Q242R as well as WT RAD51 showed protection abilities against 3′–5' exonuclease activity of the MRN complex on dsDNA in our biochemical assay (Fig. 5), so there could be different molecular mechanisms causing FA-related defects in these mutant cells. Further *in vivo* studies are required to understand the role of RAD51 in perturbing ICL repair in Q242R patient cells.

## Experimental procedures

### Plasmid DNA

The pET15b-His-thrombin-hRAD51-Q242R plasmid was generated using pET15b-His-thrombin-hRAD51 plasmid (generous gift of Dr Kurumizaka). Briefly, using two sets of primers at the Q242R mutation site (forward primer: GCTTT CAGCC AGGCG GATGC ACTTG; reverse primer: CAAGT GCATC CGCCT GGCTG AAAGC) and within the *bla* gene (forward primer: TGCCT CACTG ATTAA GCATT GGTAA; reverse primer: 5′ TTACC AATGC TTAAT CAGTG AGGCA 3′), two fragments were amplified and then ligated by Gibson assembly [New England Biolabs (NEB)].

### Oligo DNA for assays

5′-Cy5-OL2:

AAATCAATCTAAAGTATATATGAGTAAACTTGGTCTGACGTTACCAATGCTTAATCAGTGAGGCACCTATCTCAGCGATCTGTCTATTT.

5′-Cy5-OL4-40mer:

TAATACAAAATAAGTAAATGAATAAACAGAGAAAATAAAG.

5′-OL5-40mer:

CTTTATTTTCTCTGTTTATTCATTTACTTATTTTGTATTA.

Oligo DNA has been used elsewhere (45) and has been renamed in this paper. They were purchased from IDT.

### Circular ssDNA and dsDNA for assays

phiX174 ssDNA was purchased from NEB (#N3023S). The supercoiled pBluescript SK- plasmid DNA used in the D-loop assay was purified using a DNA extraction kit (QIAGEN). The supercoiled phiX174 RFI plasmid used for the ternary complex formation assay was purchased from NEB (#N3021S).

### Proteins

Human RAD51 and RAD51-Q242R plasmids were purified as described previously with minor modifications (14). Briefly, pET15b-His-RAD51 plasmid was introduced into *E.coli* BL21(DE3) codon plus and the protein expression was induced by the addition of 1 mM isopropyl β-D-1-thiogalactopyranoside. Cell extracts were prepared by disrupting cells in a lysis buffer [50 mM Tris-HCl pH 8.0, 0.5 M NaCl, 10% glycerol, 5 mM imidazole, 5 mM 2-mercaptoethanol (2-ME)]. Cell extracts were then loaded onto a HisTrap column (GE Healthcare) equilibrated with buffer A (50 mM Tris-HCl pH 8.0, 0.5 M NaCl, 10% glycerol, 5 mM imidazole, 5 mM 2-ME). His-RAD51 was eluted from the column by a linear gradient of imidazole from 5 mM to 1 M. Protein fractions were dialyzed against buffer B [50 mM Tris-HCl pH 8.0, 200 mM KCl, 0.25 mM ethylenediaminetetraacetic acid (EDTA), 10% glycerol, 2 mM 2-ME] overnight at 4 °C, and the His-tag was removed by Thrombin protease according to the manufacturer’s instruction manual (Cytiva). Protein fractions were again dialyzed by buffer C (100 mM Tris-acetate pH 7.5, 7 mM spermidine-HCl, 5% glycerol, 0.1 mM DTT) overnight at 4 °C. The proteins were precipitated by centrifugation and then resolved in buffer D (100 mM K-phosphate pH 7.0, 150 mM NaCl, 1 mM EDTA pH 8.0, 10% glycerol, 2 mM 2-ME). RAD51 was further purified by a HiTrap Q column (GE Healthcare) in buffer E (50 mM Tris-HCl pH 8.0, 0.2 M KCl, 10% glycerol, 2 mM 2-ME) using stepwise concentrations of KCl (0.2, 0.6, and 1 M). Protein fractions were further dialyzed against RAD51 storage buffer (20 mM Hepes-NaOH pH 7.5, 150 mM NaCl, 10% glycerol, 2 mM 2-ME). RAD51-Q242R was purified by the same procedure. Protein solubilities were analyzed using mass photometry analysis (Fig. S5). Human MRN was purified as previously described (46).

### ATPase assay

The ATPase assay was performed using a malachite green phosphate assay kit (POMG-25H, Bioassay Systems). Reaction mixture (10 μl) containing a buffer [20 mM Hepes-NaOH pH 7.5, 0.1 mg/ml bovine serum albumin (BSA), 1 mM DTT supplemented with 1 mM ATP and 2 mM MgCl_2_] was mixed with RAD51 together with 20.5 μM (in nt) phiX ssDNA (NEB, #N3023S). After the incubation for indicated times at 37 °C, reactions were terminated, and the absorbance at 620 nm was determined using Nanodrop (Scrum) according to the manufacturer’s instructions.

### DNA-binding assay

For ssDNA-binding assay, 2.7 μM (in nt) 5′Cy5-labeled 90-mer ssDNA Cy5-OL2 was incubated with indicated concentrations of RAD51 in a reaction mixture (10 μl) containing 20 mM hepes-NaOH pH 7.5, 0.1 mg/ml BSA, 1 mM DTT, 10 mM MgCl_2_, 150 mM KCl, and in the presence of 2 mM ATP or AMP-PNP or in the absence of any nucleotide. Reaction mixtures were incubated at 37 °C for 10 min and analyzed by 8% native polyacrylamide gel electrophoresis (PAGE) with 1 × TBE. Cy5-labeled DNA was visualized using LAS4000 (GE Healthcare) and quantified using ImageQuant TL (GE Healthcare). The dsDNA-binding assay was carried out using the same procedure as that of the ssDNA-binding assay, except that Cy5-labeled 40-mer dsDNA (Cy5-OL4 annealed with OL5) was used instead of ssDNA.

### ExoI nuclease protection assay

WT RAD51 or RAD51-Q242R (1 μM) was preincubated with Cy5-OL2 (1.35 μM in nt) in a reaction mixture (10 μl) containing 20 mM Hepes-NaOH pH 7.5, 50 mM KCl, 0.1 mg/ml BSA, 1 mM DTT, 10 mM MgCl_2_, and 2 mM ATP (or ADP, AMP-PNP) at 37 °C for 10 min. Exonuclease I (ExoI; Takara) was added to the reaction mixture to the final concentration of 0.1 units/μl and further incubated for 10 min at 37 °C. The reaction was terminated by the addition of a 2.5 μl of stop solution containing 0.625% sodium dodecyl sulfate (SDS), 200 mM EDTA, and 10 μg of Proteinase K followed by incubation at 37 °C for 15 min. The reaction mixtures were then analyzed by 8% native PAGE. Cy5-labeled DNA was visualized using LAS4000 (GE Healthcare) and quantified using ImageQuant TL (GE Healthcare).

### MRN nuclease protection assay

WT RAD51 or RAD51-Q242R (0.075–0.3 μM) was preincubated with 40-mer dsDNA (Cy5-OL4 annealed with OL5, 0.1 μM in bp) in a reaction mixture (10 μl) containing 25 mM Tris-HCl pH 7.5, 8% glycerol, 1 mM DTT, 0.2 mg/ml BSA, 1 mM MnCl_2_, 5 mM MgCl_2_, and 1 mM ATP at 37 °C for 5 min. MRN was added to indicated lanes at a final concentration of 9 nM and further incubated for 30 min at 37 °C. The reaction was terminated by the addition of a 2.5 μl of stop solution containing 0.625% SDS, 200 mM EDTA, and 10 μg of Proteinase K followed by incubation at 37 °C for 15 min. The reaction mixtures were then analyzed using 10% denaturing PAGE containing 8M urea. Cy5-labeled DNA was visualized using LAS4000 (GE Healthcare) and quantified using ImageQuant TL (GE Healthcare).

### D-loop assay

Cy5-OL2 and pBluescript were used as described previously (45) in the D-loop assay as ssDNA and its homologous dsDNA, respectively. Cy5-OL2 (0.67 μM in nt) was preincubated with indicated amounts of proteins at 37 °C for 5 min in 10 μl of buffer containing 25 mM Tris-HCl (pH 7.5), 1 mM MgCl_2_, 100 mM KCl, and 1 mM of ATP or AMP-PNP. Supercoiled pBluescript plasmid DNA was added at a final concentration of 50 μM (in bp) and further incubated at 37 °C for 10 min. The reactions were terminated by the addition of SDS-proteinase K stop solution (to the final concentration of 0.25% SDS and 0.5 mg/ml proteinase K) and further incubated at 37 °C for 5 min. The products were analyzed by electrophoresis using Orange G loading dye and 0.9% agarose gel with 1 × TAE buffer. Cy5-labeled DNA was visualized using LAS4000 (GE Healthcare).

### Ternary complex formation assay

Ternary complex formation assay was performed as previously described with minor modifications (47). The reactions were performed as described above in D-loop assay using 1.5 μM of WT RAD51 or RAD51-Q242R, and pBluescript plasmid DNA or phiX RFI (175 μM in bp) was used in the indicated reaction. Instead of SDS-proteinase K stop solution, glutaraldehyde was added to a final concentration of 0.018%, and the reaction mixture was further incubated at 37 °C for 5 min. The nucleoprotein complexes were separated by 0.9 % agarose gel electrophoresis in 1 × TAE buffer. Cy5-labeled DNA was visualized using LAS4000 (GE Healthcare).

### Mass photometry analysis

WT RAD51 and RAD51-Q242R were analyzed in the buffer containing 20 mM Tris-pH 7.5, 150 mM KCl, 10 mM MgCl_2_, and 1 mM ATP at a final concentration of 114 nM using Refeyn One mass photometer (Refeyn Ltd), as reported previously (48). The glass slides and buffer gaskets were washed with 100% ethanol and distilled water and dried under an air stream. The molecular weight of each protein was measured from 3000-frame movies according to the manufacturer’s instructions using AcquireMP and DiscoverMP softwares (Refeyn Ltd). Thyroglobulin and aldolase were used as molecular weight markers for the calibration. The histogram in the figure was created using the AcquireMP software.

## Data availability

All data are included in the article and in the Supporting information. The raw data are provided upon request.

## Supporting information

This article contains Supporting information.

## Conflict of interest

The authors declare that they have no conflicts of interest with the contents of this article.

## Supporting information

Supplemental Fig. 1 **Comparison of ssDNA- or dsDNA-binding activities of WT RAD51 and RAD51-Q242R in the presence of ATP or AMP-PNP or absence of a nucleotide.***A* and *B*, DNA-binding assays using indicated concentration of WT RAD51 and RAD51-Q242R to ssDNA (*A*) and to dsDNA (*B*) in the absence of a nucleotide. The percentage of DNA–protein complex relative to the total DNA was calculated based on the quantification of band intensities of unbound DNA (*bottom*). *C* and *D*, all DNA-binding data in Figures 2 and S1, *A* and *B* are the same but differently combined for comparison. Quantification of DNA-binding activity in the presence of ssDNA is shown in (*C*). Quantification of DNA-binding activity in the presence of dsDNA is shown in (*D*). dsDNA, double-stranded DNA; ssDNA, single-stranded DNA.

Supplemental Fig. 2 **RAD51-Q242R does not show D-loop formation ability by itself.***A*, D-loop assay. Increasing concentration (1-3 μM) of WT RAD51 and RAD51-Q242R were used in the presence of AMP-PNP. RAD51-Q242R did not mediate D-loop formation even at the highest concentration (3 μM). *B*, D-loop assay was performed in the presence of 1 mM ATP, 1 mM Mg^2+^, and 2 mM Ca^2+^ ions. Q242R did not efficiently mediate D-loop formation under these conditions. D-loop, displacement loop.

Supplemental Fig. 3 **Ternary complex formation assay.***A*, the ternary complex assay was performed in the same manner as that used to produce the results shown in Figure 7. The products were deproteinized by proteinase K treatment to determine the D-loop products. For WT RAD51, a slower migrating band corresponding to the ternary complex remains observed, indicating that it contained strand-exchange products. *B*, addition of phiX ssDNA resulted in the dissociation of RAD51-Q242R from Cy5-labeled ssDNA, showing that RAD51-Q242R/ssDNA becomes unstable when competitor dsDNA or ssDNA is involved in the reaction. dsDNA, double-stranded DNA; ssDNA, single-stranded DNA.

Supplemental Fig. 4 **DNA-binding assay under the different salt concentrations.***A*, ssDNA-binding assay using 1 μM WT RAD51 and RAD51-Q242R under the indicated KCl concentrations were carried out. *B*, the percentage of DNA–protein complex relative to total DNA was calculated based on the quantification of the band intensities of unbound DNA as shown in (*B*). ssDNA, single-stranded DNA.

Supplemental Fig. 5 **Protein solubility analyzed by mass photometry**. Mass photometry experiments using WT RAD51 and RAD51-Q242R were performed, and the data were analyzed using DiscoverMP software (Refeyn Ltd). Molecular weight distributions for WT RAD51 (*blue*, peaks at 102 and 257 kDa) and for RAD51-Q242R (*orange*, peaks at 112 and 317 kDa) are presented as a histogram created by DiscoverMP software.
