# Supplementary figures and images for: Fanconi anemia-associated mutation in RAD51 compromises the coordinated action of DNA-binding and ATPase activities
Source: J Biol Chem. 2023 Nov 2;299(12):105424. doi: 10.1016/j.jbc.2023.105424 (PMC10716581; doi:10.1016/j.jbc.2023.105424)

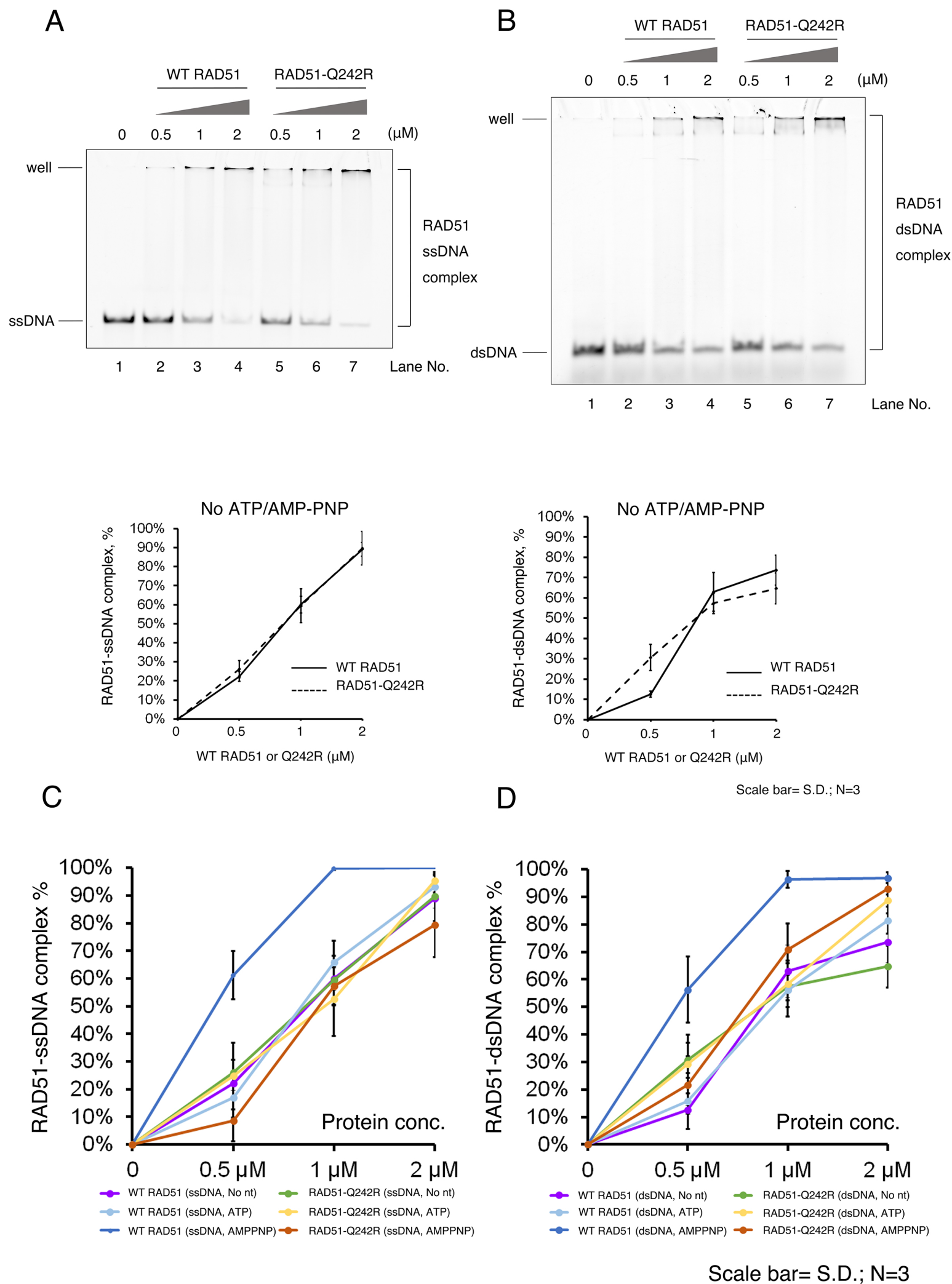

Supplement: Supplemental Fig. 1 — Comparison of ssDNA- or dsDNA-binding activities of WT RAD51 and RAD51-Q242R in the presence of ATP or AMP-PNP or absence of a nucleotide.A and B, DNA-binding assays using indicated concentration of WT RAD51 and RAD51-Q242R to ssDNA (A) and to dsDNA (B) in the absence of a nucleotide. The percentage of DNA–protein complex relative to the total DNA was calculated based on the quantification of band intensities of unbound DNA (bottom). C and D, all DNA-binding data in Figures 2 and S1, A and B are the same but differently combined for comparison. Quantification of DNA-binding activity in the presence of ssDNA is shown in (C). Quantification of DNA-binding activity in the presence of dsDNA is shown in (D). dsDNA, double-stranded DNA; ssDNA, single-stranded DNA. [file mmc1.pdf]

A

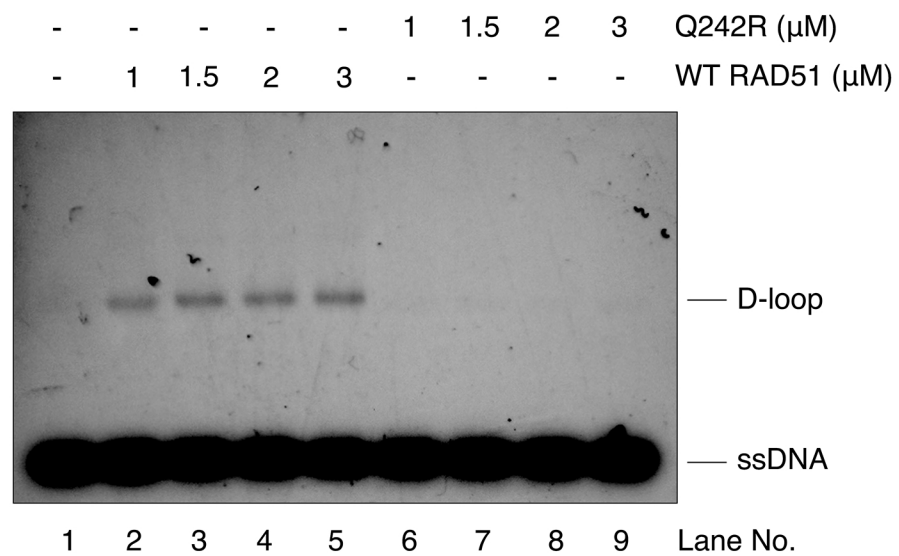

B

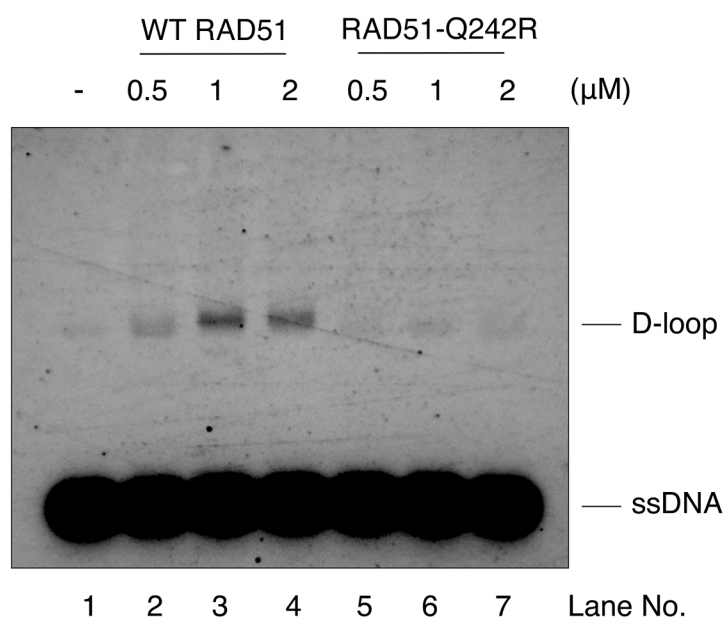

Supplement: Supplemental Fig. 2 — RAD51-Q242R does not show D-loop formation ability by itself.A, D-loop assay. Increasing concentration (1-3 μM) of WT RAD51 and RAD51-Q242R were used in the presence of AMP-PNP. RAD51-Q242R did not mediate D-loop formation even at the highest concentration (3 μM). B, D-loop assay was performed in the presence of 1 mM ATP, 1 mM Mg2+, and 2 mM Ca2+ ions. Q242R did not efficiently mediate D-loop formation under these conditions. D-loop, displacement loop. [file mmc2.pdf]

A

|   |   |     |     |     |     |                     |
|---|---|-----|-----|-----|-----|---------------------|
| - | - | 1.5 | 1.5 | -   | -   | WT RAD51 ( $\mu$ M) |
| - | - | -   | -   | 1.5 | 1.5 | Q242R ( $\mu$ M)    |
| + | + | +   | +   | +   | +   | Cy5-OL2-90 mer      |
| - | + | +   | -   | +   | -   | pBluescript         |

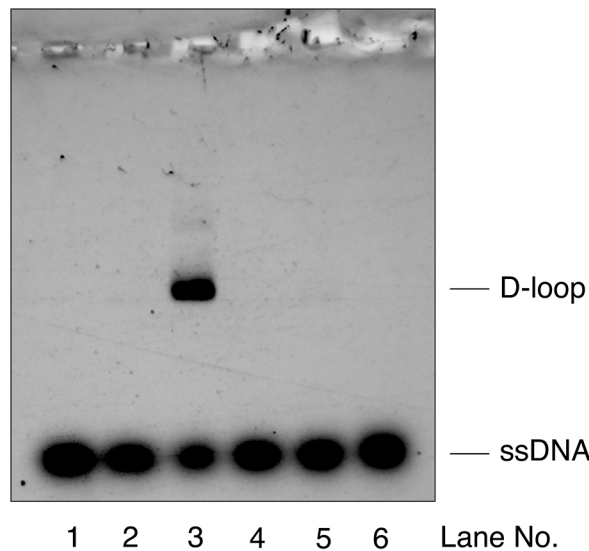

B

|   |   |   |   |     |     |     |     |                  |
|---|---|---|---|-----|-----|-----|-----|------------------|
| - | - | - | - | 1.5 | 1.5 | 1.5 | 1.5 | Q242R ( $\mu$ M) |
| + | + | + | + | +   | +   | +   | +   | Cy5-OL2-90 mer   |
| - | + | - | - | -   | +   | -   | -   | pBluescript      |
| - | - | + | - | -   | -   | +   | -   | phiX RFI         |
| - | - | - | + | -   | -   | -   | +   | phiX ssDNA       |

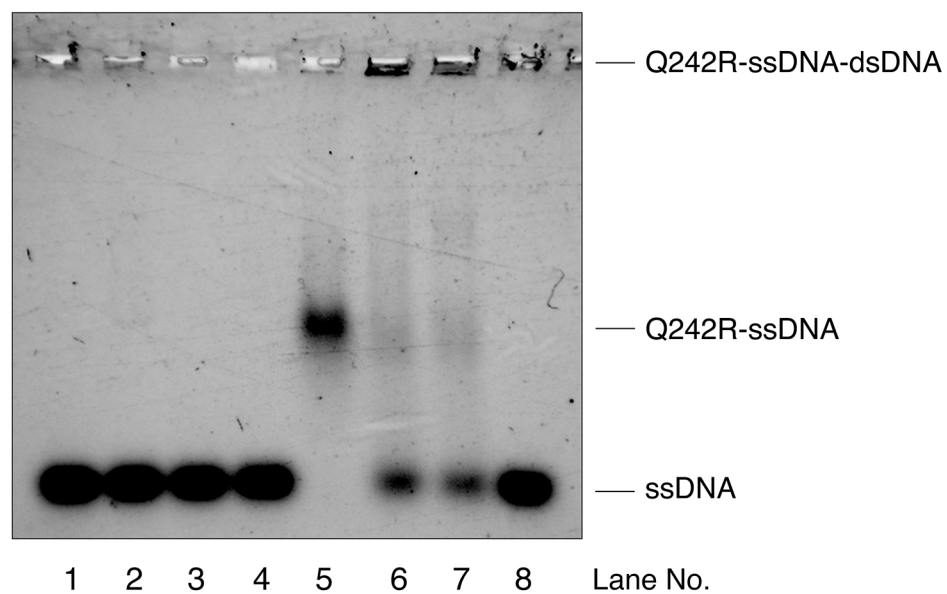

Supplement: Supplemental Fig. 3 — Ternary complex formation assay.A, the ternary complex assay was performed in the same manner as that used to produce the results shown in Figure 7. The products were deproteinized by proteinase K treatment to determine the D-loop products. For WT RAD51, a slower migrating band corresponding to the ternary complex remains observed, indicating that it contained strand-exchange products. B, addition of phiX ssDNA resulted in the dissociation of RAD51-Q242R from Cy5-labeled ssDNA, showing that RAD51-Q242R/ssDNA becomes unstable when competitor dsDNA or ssDNA is involved in the reaction. dsDNA, double-stranded DNA; ssDNA, single-stranded DNA. [file mmc3.pdf]

A

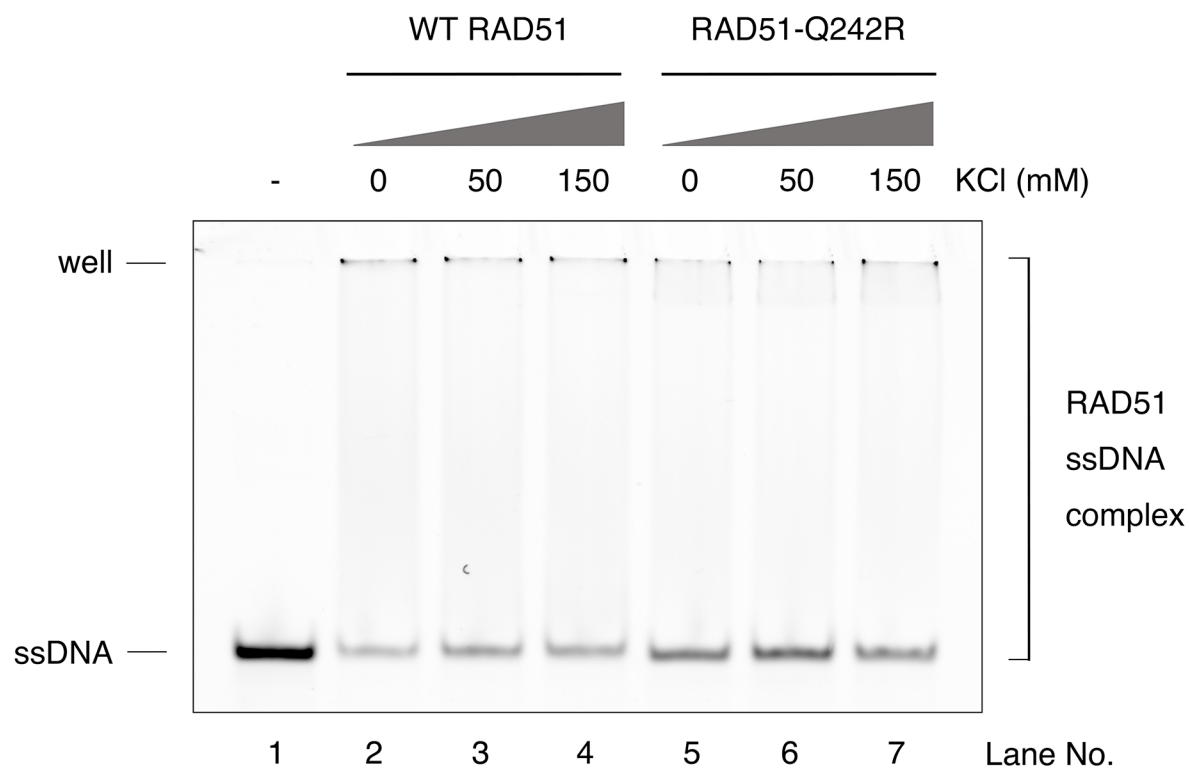

B

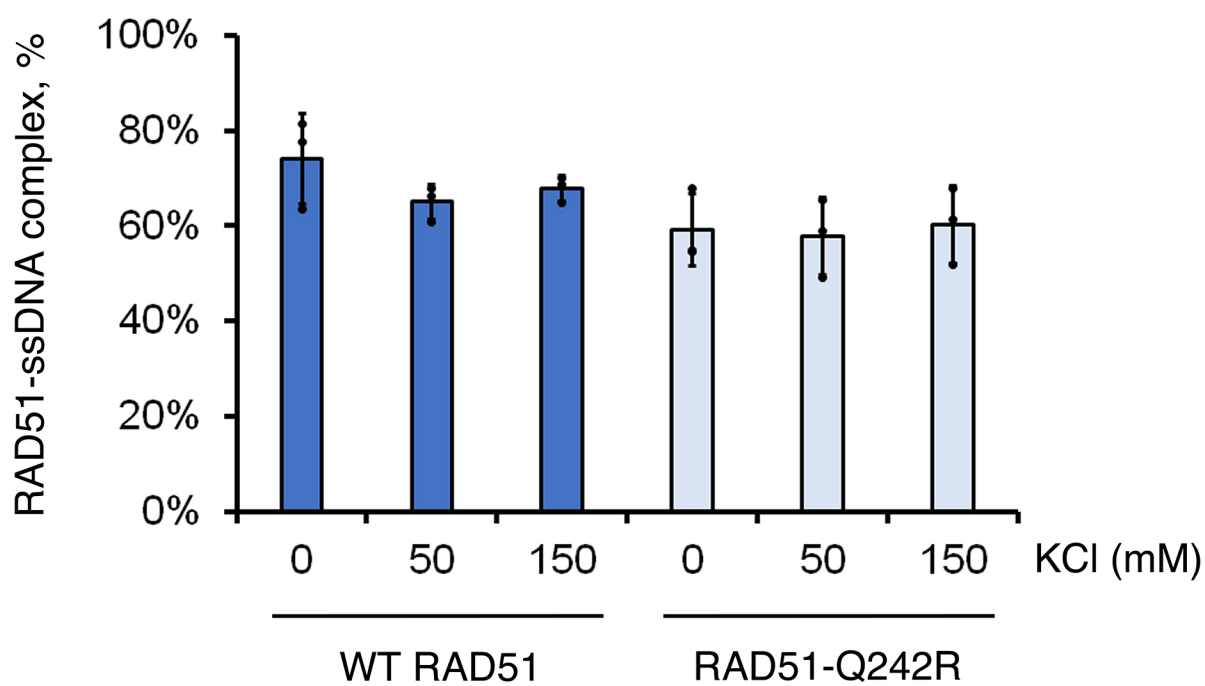

Scale bar= S.D.; N=3

Supplement: Supplemental Fig. 4 — DNA-binding assay under the different salt concentrations.A, ssDNA-binding assay using 1 μM WT RAD51 and RAD51-Q242R under the indicated KCl concentrations were carried out. B, the percentage of DNA–protein complex relative to total DNA was calculated based on the quantification of the band intensities of unbound DNA as shown in (B). ssDNA, single-stranded DNA. [file mmc4.pdf]

A

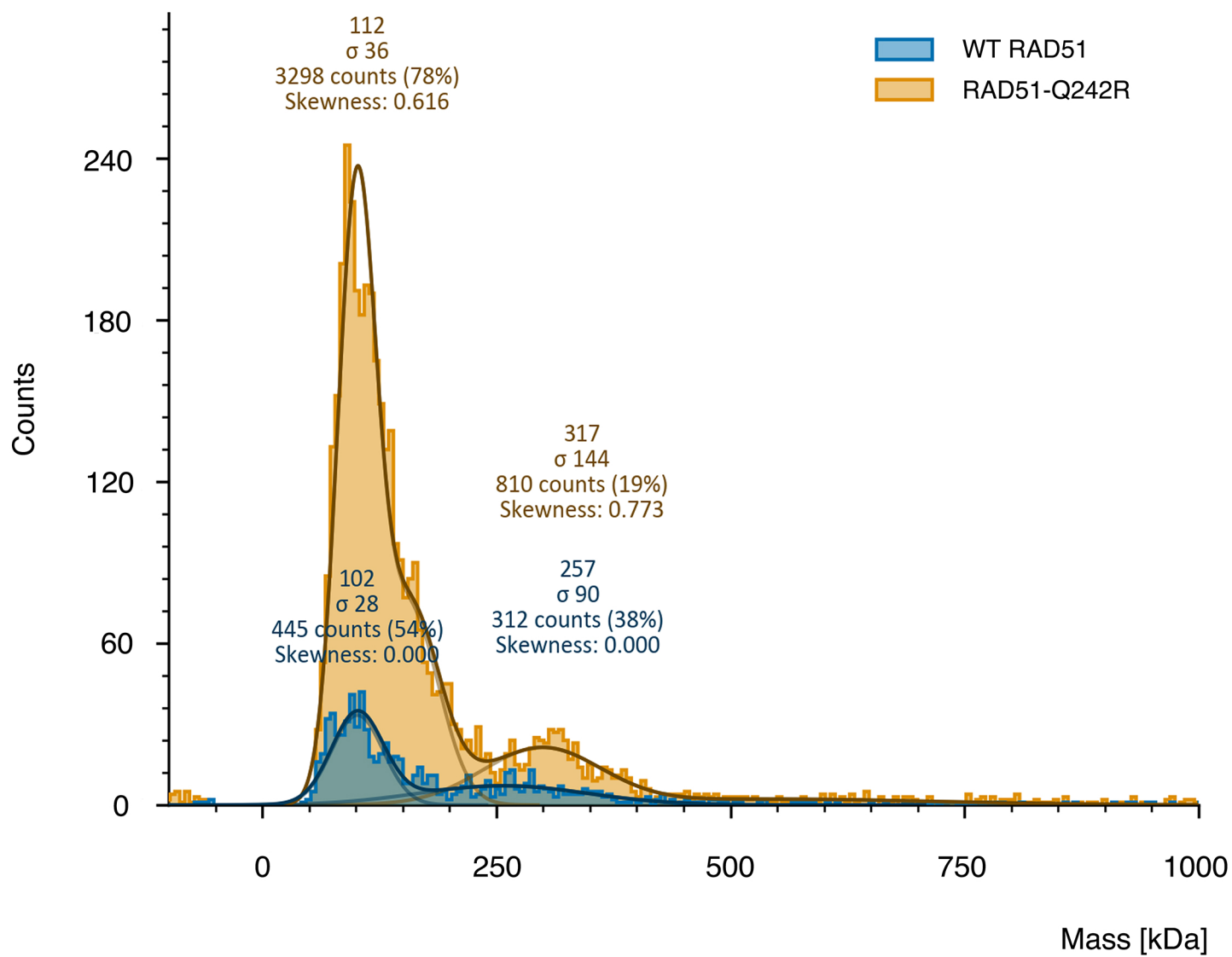

Supplement: Supplemental Fig. 5 — Protein solubility analyzed by mass photometry. Mass photometry experiments using WT RAD51 and RAD51-Q242R were performed, and the data were analyzed using DiscoverMP software (Refeyn Ltd). Molecular weight distributions for WT RAD51 (blue, peaks at 102 and 257 kDa) and for RAD51-Q242R (orange, peaks at 112 and 317 kDa) are presented as a histogram created by DiscoverMP software. [file mmc5.pdf]
